# Knowledge‐based planning for intensity‐modulated radiation therapy: A review of data‐driven approaches

**DOI:** 10.1002/mp.13526

**Published:** 2019-04-24

**Authors:** Yaorong Ge, Q. Jackie Wu

**Affiliations:** ^1^ Department of Software and Information Systems University of North Carolina at Charlotte Charlotte NC 28223 USA; ^2^ Department of Radiation Oncology Duke University Medical Center Durham NC 27710 USA

**Keywords:** IMRT, IMRT planning, intensity‐modulated radiation therapy, KBP, knowledge modeling, knowledge‐based planning, machine learning, tomotherapy, VMAT

## Abstract

**Purpose:**

Intensity‐Modulated Radiation Therapy (IMRT), including its variations (including IMRT, Volumetric Arc Therapy (VMAT), and Tomotherapy), is a widely used and critically important technology for cancer treatment. It is a knowledge‐intensive technology due not only to its own technical complexity, but also to the inherently conflicting nature of maximizing tumor control while minimizing normal organ damage. As IMRT experience and especially the carefully designed clinical plan data are accumulated during the past two decades, a new set of methods commonly termed knowledge‐based planning (KBP) have been developed that aim to improve the quality and efficiency of IMRT planning by learning from the database of past clinical plans. Some of this development has led to commercial products recently that allowed the investigation of KBP in numerous clinical applications. In this literature review, we will attempt to present a summary of published methods of knowledge‐based approaches in IMRT and recent clinical validation results.

**Methods:**

In March 2018, a literature search was conducted in the NIH Medline database using the PubMed interface to identify publications that describe methods and validations related to KBP in IMRT including variations such as VMAT and Tomotherapy. The search criteria were designed to have a broad scope to capture relevant results with high sensitivity. The authors filtered down the search results according to a predefined selection criteria by reviewing the titles and abstracts first and then by reviewing the full text. A few papers were added to the list based on the references of the reviewed papers. The final set of papers was reviewed and summarized here.

**Results:**

The initial search yielded a total of 740 articles. A careful review of the titles, abstracts, and eventually the full text and then adding relevant articles from reviewing the references resulted in a final list of 73 articles published between 2011 and early 2018. These articles described methods for developing knowledge models for predicting such parameters as dosimetric and dose‐volume points, voxel‐level doses, and objective function weights that improve or automate IMRT planning for various cancer sites, addressing different clinical and quality assurance needs, and using a variety of machine learning approaches. A number of articles reported carefully designed clinical studies that assessed the performance of KBP models in realistic clinical applications. Overwhelming majority of the studies demonstrated the benefits of KBP in achieving comparable and often improved quality of IMRT planning while reducing planning time and plan quality variation.

**Conclusions:**

The number of KBP‐related studies has been steadily increasing since 2011 indicating a growing interest in applying this approach to clinical applications. Validation studies have generally shown KBP to produce plans with quality comparable to expert planners while reducing the time and efforts to generate plans. However, current studies are mostly retrospective and leverage relatively small datasets. Larger datasets collected through multi‐institutional collaboration will enable the development of more advanced models to further improve the performance of KBP in complex clinical cases. Prospective studies will be an important next step toward widespread adoption of this exciting technology.

## 1 Introduction

Radiation therapy is a widely adopted and effective cancer treatment that leverages highly advanced and complex technologies. With the advent of intensity‐modulated radiation therapy (IMRT), physicians have a tremendous opportunity to maximize cancer control while minimizing toxicity to normal organs. However, achieving this inherently contradicting goal using IMRT requires significant knowledge, experience, and time due to the complexity of technologies and the limitation in our understanding of patient conditions. We note that the IMRT technology has led to a number of different implementations in recent years including Volumetric Arc Therapy (VMAT) and Tomotherapy. In the remainder of this paper, the term “IMRT” by itself will generally refer to all variations of IMRT implementations. When it is listed together with VMAT and/or Tomotherapy, it refers specifically to the original implementation.

To tackle the challenges in radiation therapy, knowledge‐based systems have been developed as early as 1980s to aid the design of radiation treatment plans.^1^, ^2^ The knowledge‐based systems reported during that period refer mainly to expert‐based systems that aim to capture clinician knowledge and experience in terms of rules and algorithms. These rule‐based approaches in recent years have led to a type of system that is commonly called “automatic (or automated) planning systems” (e.g., Ref. 3, 4, 5). These systems aim to encode sophisticated planning knowledge into complex and often iterative algorithms to generate clinically acceptable IMRT plans automatically. Note that these automatic planning systems are not data‐driven in the sense that their main algorithms do not rely on predictive models that are based on a database of prior planning data.

As IMRT experience and especially the carefully designed clinical plans are accumulated over the past two decades, a new set of data‐driven methods has been developed in recent years with an aim to improve the quality and efficiency of IMRT planning by learning from the past high‐quality clinical plans. The term “knowledge‐based planning” (KBP) or simply KBP has now frequently been used to refer to this specific class of data‐driven approaches to IMRT planning. Some of this development has led to commercial products recently and allowed the investigation of KBP in numerous clinical applications. This has somewhat solidified the narrower definition of KBP that draws knowledge from only one source, the database of prior clinical plan data, and assume that other sources of knowledge, such as treatment trade‐off and clinician experience, are embedded in the design of prior clinical plans.

In this literature review, we will focus on KBP methods that are data‐driven. We will not include the types of KBP methods, such as automatic planning systems, that do not rely on models and prior clinical plans. We will attempt to present a summary of this specific class of data‐driven KBP methods and recent clinical validation results. We will slightly broaden the definition a bit to include any data‐driven method that aims to improve IMRT planning in some aspects that do not necessarily lead to complete final plans. For example, we will include studies that learn from prior plan data to predict or generate beam configurations, objective function priorities, or some specific dose metric in one of the organs at risk, or to identify unacceptable plans in the quality assurance process. By reviewing the prediction targets, modeling methods, data sources, application areas, and validation results, we aim to present a clear understanding of the state‐of‐the‐art of the data‐driven KBP approach and summarize the performance of current methods in comparison to manual planning process. We hope that this exercise will also help us gain insights into potential gaps in the current approaches that warrant further research.

## 2 Materials and methods

Even though this review focuses on the methods and technical validation of KBP rather than patient outcomes, wherever appropriate, we follow the guidelines stated in The PRISMA Statement for Reporting Systematic Reviews and Meta‐Analyses of Studies That Evaluate Health Care Interventions.^6^

### 2.A. Article search

To identify relevant articles for KBP, we conducted searches in the NIH Medline database in March 2018 using the PubMed interface. We did not use any time constraints for this search and included only articles published in journals and written in English. We started with keywords that identify knowledge, radiation therapy, planning and expanded the search to include variations of keywords related to these concepts. In addition, we included keywords in the abstracts that indicate the use of a set of prior plans. The final search string is: (atlas[Title] OR reasoning[Title] OR model[Title] OR models[Title] OR modeling[Title] OR learning[Title] OR prediction[Title] OR predicting[Title] OR feature[Title] OR quantitative analysis[Title] OR factor analysis[Title] OR identification[Title] OR knowledge[Title] OR automated[Title] OR automate[Title] OR automatic[Title] OR semiautomated[Title] OR semi‐automated[Title]) AND (IMRT[Title] OR VMAT[Title] OR SBRT[Title] OR treatment[Title] OR therapy[Title] OR radiotherapy[Title] OR tomotherapy[Title]) AND (beam angle[Title] OR dose[Title] OR quality[Title] OR QA[Title] OR plan[Title] OR planning[Title] OR sparing[Title] OR optimization[Title] OR objective function[Title]) AND (plans[Title/Abstract] OR dataset[Title/Abstract] OR cases[Title/Abstract] OR patients[Title/Abstract]) AND English[lang].

### 2.B. Article eligibility criteria

Articles were included in this review if they satisfied the following criteria:

1. Describing or validating methods for improving some aspects of radiation therapy planning. These aspects can include reference plans, dosimetric parameters, dose‐volume histogram, voxel‐level doses, objective function weights/optimization priorities, beam configurations, model hyper‐parameters, and quality assurance metrics. Outcomes studies and other studies not related to planning are excluded.
2. Focusing on external beam radiation therapy, which may include various forms of IMRT (i.e., IMRT, VMAT, and Tomotherapy) of both photon and proton beams but exclude brachytherapy.
3. Employing a set of prior clinical plans as a core component of the method. Articles that use prior clinical plans to validate methods that do not rely on prior clinical plans are excluded.

### 2.C. Article selection

**Figure 1:**
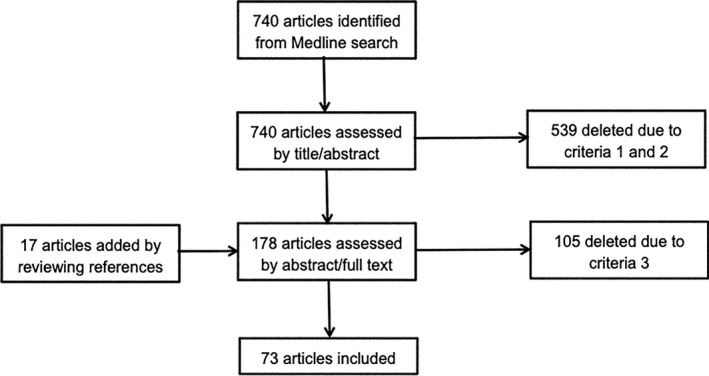
Flow diagram of article selection.

The search strategy retrieved 740 articles from the Medline database. After reviewing the title and abstract of the articles in the initial list, the authors reduced the list to 161 by filtering out articles that do not satisfy the first two eligibility criteria. We then added to this list a few additional articles based on reviewing of reference lists. The new list of 178 articles was further filtered by the third eligibility criteria by reviewing abstracts and when the abstract is not conclusive, the full text of the articles. This step resulted in the final set of 73 articles that are included in the following review (Fig. 1).

## 3 Results

**Figure 2:**
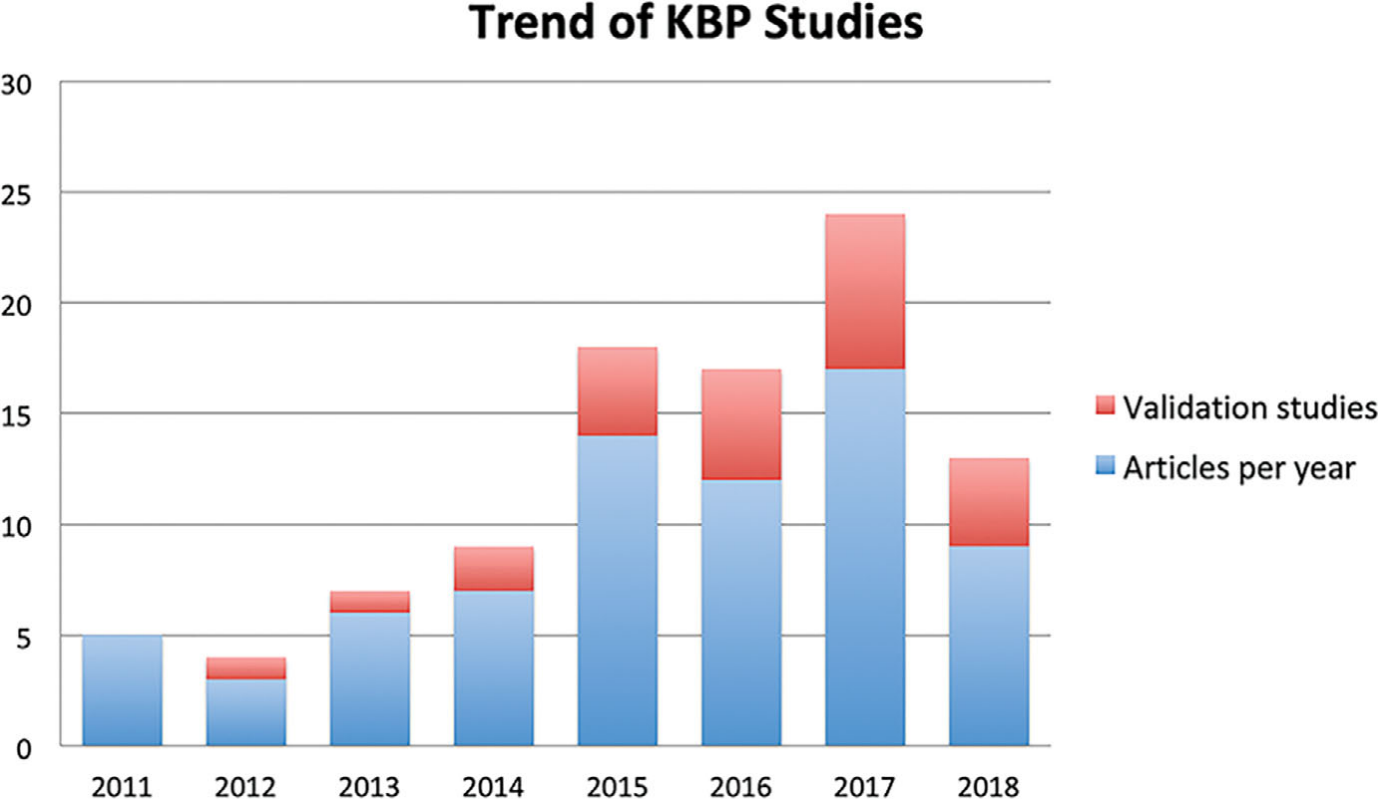
Trend of publications related to knowledge‐based planning. [Color figure can be viewed at wileyonlinelibrary.com]

The 73 KBP‐related articles included in this review were published between 2011 and early 2018. The number of studies has shown an increasing trend in recent years (Fig. 2). In fact, the number of studies in the 4 yr since 2014 accounts for more than 70% of the total articles with only a few months included in 2018.

Almost a third of the articles appeared in Medical Physics. The other top publication venues also include Journal of Applied Clinical Medical Physics, International Journal of Radiation Oncology Biology and Physics, Physics in Medicine and Biology, and Radiation Oncology (Table 1). A total of 16 journals have published KBP‐related research results.

**Table 1:** The publication venues that reported KBP studies

Journal title	Number of KBP articles
Medical Physics	23
Journal of Applied Clinical Medical Physics	9
International Journal of Radiation Oncology Biology Physics	8
Radiation Oncology	8
Physics in Medicine and Biology	8
Radiotherapy and Oncology	6
Biomedical Materials and Engineering	1
Plos One	2
Advances in Radiation Oncology	1
International Journal of Computer Assisted Radiology and Surgery	1
IEEE Transactions on Medical Imaging	1
Artificial Intelligence in Medicine	1
Medical Dosimetry	1
Physica Medica	1
Practice of Radiation Oncology	1
Frontiers in Oncology	1

KBP, knowledge‐based planning.

As shown in Table 2, a wide variety of cancer sites have been studied with the KBP methods. However, a significant number of studies have focused on prostate cancer (more than one‐third). And the three cancer sites, prostate, head and neck (H&N), and lung, accounted for more than two‐thirds of articles reviewed.

**Table 2:** The number of articles that performed studies on each cancer site

Cancer sites	Number of KBP articles
Prostate	31
Head & Neck	16
Lung	13
Breast	7
Brain	3
Cervical cancer	3
Spine	3
Esophageal cancer	2
Nasopharyngeal carcinoma	2
Rectal	2
Hepatocellular carcinoma	1
CNS, GI, Genitourinary, GYN, Pediatric	1
Glioblastoma	1
Malignant pleural mesothelioma	1
Pancreatic	1
Pelvic	1
Thoracic	1

KBP, knowledge‐based planning.

In the following sections, we summarize the 73 articles in terms of three dimensions: the purpose of KBP methods, the methods for KBP, and the performance of current KBP on major cancer types.

### 3.A. Purpose of knowledge‐based planning

Knowledge models have been created to predict a variety of variables that impact the quality and efficiency of IMRT planning. We can roughly categorize existing work into six types of variables that the models aim to predict:

1. Dose‐volume histogram (DVH) (36 articles)

This group of methods aims to predict the entire DVH curve for a new patient and then frequently uses the predicted DVHs to guide the plan optimization process 7, 8, 9, 10, 11, 12, 13, 14, 15, 16, 17, 18, 19, 20, 21, 22, 23, 24, 25, 26, 27, 28, 29, 30, 31, 32, 33, 34, 35, 36, 37, 38, 39, 40, 41, 42

- One or more specific dose metrics (14 articles)

These methods aim to predict single or a small number of dose metrics to either guide plan optimization or a specific planning decision (e.g., the need for hydrogel injection).^21^, ^43^, ^44^, ^45^, ^46^, ^47^, ^48^, ^49^, ^50^, ^51^, ^52^, ^53^, ^54^, ^55^

- Voxel‐level doses (13 articles)

This group of methods predicts dose at each voxel in 3D space.^56^, ^57^, ^58^, ^59^, ^60^, ^61^, ^62^, ^63^, ^64^, ^65^, ^66^, ^67^, ^68^ The predicted dose map is used to guide plan optimization or generate final plans directly (e.g., using dose mimicking algorithm^68^).

- Objective function weights (three articles)

There are two papers by the same group that aim to predict correct objective function weights, so that plans can be generated automatically.^69^, ^70^ A third paper examined the sample size needs for predicting objective function weights.^21^

- Beam‐related parameters (six articles)

There are a number of articles that aim to determine beam‐related parameters such as the number and angle of beams and jaw settings.^3^, ^71^, ^72^, ^73^, ^74^, ^75^

- Quality assurance metrics (three articles)

This group of methods learns from prior clinical plans to predict the quality of a new plan.^76^, ^77^, ^78^ Note that a number of quality assurance methods are based on DVH or dose‐volume parameter prediction models.^17^, ^18^, ^33^, ^34^, ^49^

### 3.B. Methods for knowledge‐based planning

Methods for KBP can be further divided into two major categories: (a) case and atlas‐based methods; and (b) statistical modeling and machine learning methods.

#### 3.B.1. Case and atlas‐based methods

The case and atlas‐based approaches aim to improve the planning of a present case by finding one or more similar cases in the database of prior clinical plans. Two components are critical in these methods: (a) a similarity measure for identifying the matching cases; and (b) a method to transfer useful knowledge from prior plans to the present case. There are twenty‐four (24) articles in this category; the similarity measures and transferred knowledge of each are summarized in Table 3.

**Table 3:** Case‐ and atlas‐based methods: similarity measure and knowledge transfer

Articles	Approach	Similarity measure	Knowledge transfer
Chanyavanich et al.^56^, ^57^, ^61^	Direct	Mutual information of the beam's eye view projections	Treatment parameters such as beam geometry and structure constraints and weights were transferred to the query case. The fluence maps were transferred after a deformable registration
Mishra et al.^43^ – Case‐based reasoning framework	Direct	Similarity is measured by both clinical variables such as clinical stage, Gleason score, and prostate‐specific antigen as well as rectum DVH values at five selected points	Dose constraints were transferred after adaptation
Petrovic et al.^74^	Direct	Further introduced knowledge‐light adaptation into the case retrieval process to improve case selection accuracy.	Dose constraints were transferred after adaptation
Wu et al.^44^, ^45^, ^46^, ^47^, ^55^	Direct	Based on the concept of OVH, which describes the fractional volume of an OAR that is within a specified distance from a PTV. For each OVH percent volume, the set of matching cases included all cases with smaller OVH values	The minimal DVH value at the percentage volume of the matching cases was transferred to the new case
Zhang et al.^3^ belongs to the automatic planning approach. We include it here because its beam selection is based on a database of prior clinical plans	Direct	Based on tumor location	Beam number and angles
Schreibmann et al.^62^, ^71^	Direct	Based on an iterative closest point registration algorithm and a score based on point to point distance	The beam settings and multileaf collimator positions for the best match were transferred to the new case
Zarepisheh et al.^14^	Direct	Based on machine learning algorithm that finds the best match of DVH curve using geometric features such as overlapping volume and mutual information	
Zhou et al.^49^	Direct	The overlap area of OVH curves as the basis for similarity	Transferred DVH of OARs and PTV as optimization constraints.
Sheng et al.^63^ – Atlas‐based method	Direct	The generation of atlases and matching of a query case to the best atlas were both based on two specially designed features, the PTV and SV concaveness angle and the percent distance (from SV) to the PTV	Treatment parameters of the atlas case were transferred
Deshpande et al.^24^	Direct	Weighted sum of three difference values, the prescription dose differences, the OVH differences, and the difference of STS, which is a four‐dimensional histogram encoding the radial distance, azimuth, and elevation of PTV in relation to the center of an OAR. The difference of histograms is calculated by the earth mover's distance	The DVHs of top matching cases were presented for reviewing
McIntosh et al.^64^, ^67^, ^68^	Indirect	Each case in the database was associated with a contextual ARF that predicts dose at each voxel based on its location and image features. Each case was also associated with a random forest (pRF) that predicted the accuracy of the ARF for a new case based on its similarity to the new case's ARF. The set of matching cases had the smallest predicted errors from the associated pRF's.	The average predicted dose at voxel level from the ARF's of the matching cases was transferred as the voxel‐level dose of the new case
Li et al.^33^ – Atlas‐based method	Direct	A single atlas FDG‐PET volume was created from a set of prior clinical volumes using deformable registration of images and averaging of intensity values	The atlas was used as a template to generate a substructure of ABM within the pelvic bone marrow with a goal to improve sparing of ABM without manual contouring of ABM.
Valdes et al.^53^	Indirect	Differences between dosimetric indices of a database case and the predicted dosimetric indices of a query case must be smaller than predetermined thresholds. The predictions were based on boosted decision trees (random forest) that use features of anatomical information, medical records, treatment intent, and radiation transport.	Dosimetric information of matching cases was displayed

ABM, active bone marrow; ARF, atlas regression forest; PTV, planning target volume; OAR, Organ at Risk; OVH, overlap volume histogram; STS, spatial target signature; SV, seminal vesicle.

We can divide the similarity measures into two general categories. The direct approach defines similarity directly based on some features of the images, structures, and clinical variables. The indirect approach uses models and features to predict dose parameters first and then use the similarity of predicted dose parameters to select matching cases. Transferred knowledge ranges from planning parameters to voxel‐level dose.

#### 3.B.2. Statistical modeling and machine learning methods

The statistical modeling and machine learning approaches attempt to create a predictive model from the database of prior clinical cases. We summarized these methods in Table 4 in terms of input features, modeling methods, and prediction outcomes.

**Table 4:** Statistical modeling and machine learning: features, models, and prediction outcomes

Articles	Input features	Modeling methods	Prediction outcomes
Zhu et al.^7^, ^9^, ^10^, ^13^, ^17^, ^20^, ^82^	Volume features: PTV‐OAR overlap volume etc. Distance features: first three PCA components of Distance‐to‐Target Histogram	Support vector regression Multivariate stepwise regression Model/regression tree	The first three PCA components of DVH
Appenzoler et al.^8^	OAR distance‐to‐PTV	Sub‐DVH as basis functions of an OAR volume function of overlap subvolumes Function fitting using least squares minimization	DVH
Lee et al.^69^, ^70^	OVH values	Logistic regression Linear regression K‐nearest neighbor	Weight for an OAR constraint (Rectum, Bladder)
Yang et al.^48^	Lx – distance from PTV that result in x% of overlap in OVH	Linear regression	Dx – dose received by x% of OAR volume
Fogliata et al. 11, 12, 15, 16, 18, 22, 23, 25, 26, 27, 28, 29, 31, 32, 34, 35, 37, 38, 39, 40, 41, 75	Volume features: PTV‐OAR overlap volume etc.Distance features: PCA components of Distance‐to‐target histogram Other unpublished features	Multivariate regression (RapidPlan)	DVH
Nwankwo et al.^59^, ^60^	Distance‐to‐PTV Slice level	Mean‐dose‐at‐distance function Mean dose standard deviation function Slice weight function	Voxel dose
Amit et al.^72^	Beam‐independent features: tumor distribution, tumor height Beam‐dependent features: tumor‐organ overlap, beam distance, tumor projection shape	Random forest regression	Beam angle
Liu et al.^58^	3D OAR structures	Active shape model Active optical flow model	Voxel dose
Wang et al.^19^	First two PCA component scores of OVH of OARs Z‐axis overlap index	Stepwise multiple regression	Mean lung dose Mean heart dose (forming a Pareto Front)
Yuan et al.^73^	Beam number and angles	K‐medoids clustering	Standard beam bouquets
Cooper et al.^50^	Distance to the tangent field edge	Logistic regression	Left anterior descending artery maximum dose
Kuo et al.^51^	Contralateral/ipsilateral lung volumes Ipsilateral normal/total lung volume MILD	Linear regression	Prescription dose MILD Prescription dose
Shiraishi et al.^65^	PTV volume Number of fields Azimuthal angle Elevation angle Distance from PTV Distance from OARs	Artificial neural network (1 hidden layer with 10 nodes)	Voxel dose
Valdes et al.^76^, ^77^	78 complexity metrics: faction of MU per dose, jaw position, etc.	Poisson regression with Lasso regularization	Passing rate
Campbell et al.^66^	Geometric features: distance‐to‐PTV, distance to OARs, etc. Plan features: target volume, photon energy, etc.	Artificial Neural Network (1 hidden layer with 25 nodes)	Voxel dose
Fan et al.^30^	Distance‐to‐PTV Angle with respect to origin of coordinate (center of CT)	KDE	DVH
Powis et al.^52^	Fractional OAR‐PTV volume overlap Prescription dose	Curve fitting	Mean rectum dose
Brown et al.^78^	Control point features Beam features Fraction group features Plan features	Ensemble‐outlier filtering Normalized cut sampling SVM	Classification (acceptable vs unacceptable plans)
Millunchick et al.^54^	Fractional overlap of parotid with combined targets, and with 0.5 and 1.0 cm margins	Stepwise regression	Parotid mean dose

PCA, principal component analysis; PTV, planning target volume; OAR, organ at risk; OVH, overlap volume histogram; MILD, mean ipsilateral lung dose; KDE, Kernel density estimate.

There are 51 articles in this category. Most methods are based on regression models such as multivariate linear regression, stepwise regression, logistic regression, Poisson regression, and support vector regression. Other methods include curve fitting, function fitting, kernel density estimation, artificial neural networks, random forest, active shape model, optical flow model, support vector machine, and clustering. An important factor of the modeling approach is the definition and selection of features. Table 4 lists the major features that are used by each model.

A number of articles describe validation results of the commercially available RapidPlan system (Varian Medical Systems, Palo Alto, CA, USA). According to Varian's company website, this system is largely inspired by the multivariate linear regression approach described by Yuan et al.^9^ In Table 4, these articles are grouped together under Fogliata et al.

### 3.C. Performance of KBP

Most studies of KBP methods provide validation results using either cross‐validation or holdout test data. Since prostate, H&N, and lung are the most studied cancer types, we summarize the outcomes of KBP methods for these three cancers in Tables 5, 6, 7 in terms of method type, test sample size, validation target, validation metric, and results for OARs and planning target volume (PTV). Note that we included in these tables only studies that used at least 10 test samples and reported validation results in comparison to clinical plans. As shown in Table 7, only three studies have more than 10 test samples and validation results comparing to clinical planning results although 13 studies have involved lung cancer planning.

**Table 5:** Performance of KBP on prostate IMRT/VMAT (studies with 10 or more test cases)

Articles	Method type	Sample size	Validation target	Validation metrics	Rectum	Bladder	Target
Chanyavanich et al^56^	Case/voxel dose	10	Re‐planned vs clinical	Percent difference: mean	D20 1.8 D30 ‐2.5 D50 ‐13.9	D20 ‐5.9 D30 ‐12.2 D50 ‐24.9	D98 ‐0.03 D95 0.62 D1 2.5
Appenzoller et al.^8^	Model/DVH	20	Predicted vs clinical	Sum of residuals: mean Restricted sum of residuals: mean	0.003 0.02	−0.008 0.013	
Yuan et al.^9^	Model/DVH	24	Predicted vs clinical	Error bound of V99,85, 50%	71% of cases within 6% of error bound	71% of cases within 6% of error bound	
Good et al.^57^	Case/voxel	55	Re‐planned vs clinical	Percent difference: mean	V75 ‐1.15* V65 ‐4.10* V40 ‐11.97*	V75 ‐0.48 V65 ‐1.18* V40 ‐2.18	HI ‐2.8* D1 ‐2.5*
Nwankwo et al.^59^	Case/voxel	33	Predicted vs clinical	Mean voxel dose difference (magnitude)	0.23 – 8.22	0.26 – 12.19	
Nwankwo et al.^60^	Case/voxel	30	Re‐planned vs clinical	Mean difference	D10 3.0* D30 5.6* D50 2.4 D70 ‐0.3 D90 ‐0.7	D10 0.1 D30 ‐3.0* D50 ‐2.7 D70 0.0 D90 1.0	D05, D95, UI =
Sheng et al.^63^	Atlas/voxel	20	Re‐planned vs clinical	Mean difference	gEUD ++* V65 = V100 + +*	gEUD ++* V65 + +* V100 + +*	CI ++* HI =
Yang et al.^20^	Model/DVH	10	Re‐planned vs clinical	Percent difference	Dmax ++0.14% D10 cc ++2.11% D17 + +2.72% D40 + +0.27%	Dmax –0.46% D10 cc –<1.54% D25 + +0.69% D40 + +0.81%	D98 = <2.31% Dmax –0.06%
Boutilier et al.^21^	Model/DVH	100	Predicted vs clinical	Absolute difference	D30 ~10 D50 ~7	D30 ~7 D50 ~3	
Hussein et al.^25^	RapidPlan	10	Re‐planned vs clinical	Mean difference	V30 ‐0.8 V50 ‐3.1* V70 ‐0.4 D1 cm ‐0.3*	V50 ‐3.5 V75 ‐0.2 D1 cm 0.0	PTV High D98 0.1 D2 0.7* PTV Inter D98 ‐0.2 D2 0.3* PTV Low CI ‐0.1* D98 0.8* D2 ‐1.2*
Cagni et al.^27^	RapidPlan	20	Re‐planned vs clinical	Percent differences	Dmean ‐1.66* V20 ‐6.32* V50 ‐1.03 V60 0.54 V65 3.71* V70 0.55 NTCP 3.02*	Dmean 0.52 V20 0.93 V60 0.61 V70 0.15 NTCP 3.72*	D98 0.63* D50 0.44 D2 1.09* HI 2.03* CI 5.02
Masi et al.^34^	RapidPlan	10	Re‐planned vs clinical	Mean difference	Dmean ‐3.6* Max to 0.1 cc 0.2* V70 ‐0.8 V65 ‐1.5 V50 ‐4.0*	Dmean ‐3.9* V75 ‐1.8* V70 ‐0.6 V65 ‐2.9*	D95 ‐0.1 Dmax 0.3 HI5% ‐0.01* HI1% ‐0.01
Schubert et al.^35^	RapidPlan	60	Re‐planned vs clinical	Mean difference	Dmean 0.9* D1% ‐0.4* V40 0.0 V45 ‐2.4* V50 1.6	Dmean 0.6* D1% 0.1 V40 ‐2.5* V45 ‐1.1 V50 1.9*	Dmean 0.0 D2% 0.2 D98 0.0 HI 0.0 CI 0.0
Wall et al.^55^	Case/DVH indices	31	Re‐planned vs clinical	Mean difference	Dmean ‐9.41	Dmean ‐7.81	V98 = V100 = Dmean = Dmax =
Zhang et al.^42^	Model/DVH	111	Predicted vs clinical	Weighted root mean square error of DVH	~3%	~3%	

KBP, knowledge‐based planning; IMRT, intensity‐modulated radiation therapy; VMAT, volumetric arc therapy.

The difference direction is “KBP ‐ Clinical”. Thus, negative values mean KBP value is smaller. Where no value is provided, ++ indicates better metrics, – indicates worse metrics, = indicates similar metrics. The sign * means the metric is statistically significant with a *P*‐value < 0.05. The sign ~ indicates the value is estimated from a graph.

**Table 6:** Performance of KBP on H&N IMRT/VMAT (studies with 10 or more test cases)

Articles	Method type	Sample size	Validation target	Validation metrics	OARs	Target
Wu et al.^44^	Case/DVH	15	Re‐planned vs clinical	Mean difference	Cord + 4 D0.1 cc ‐6.9 Bstem D0.1 cc ‐7.7 Cparotid V30 ‐8.7	
Wu et al.^45^	Case/DVH	40	Re‐planned vs clinical	Mean difference	Cord + 4 D0.1 cc ‐1.68 Bstem D0.1 cc ‐2.77 Esoph D1 cc 1.52 I‐inn ear Dmean ‐3.65 C‐inn ear Dmean ‐4.83	PTV70 V95 0.31 PTV63 V95 0.4 PTV70 D5‐95 ‐0.9 Ring63 D5‐95 ‐0.87 Ring58.1 D5‐95 ‐1.61
Yuan et al.^9^	Model/DVH	24	Predicted vs clinical	Error bound of parotid mean dose	63% cases are within 6% error bound	
Lian et al.^10^ – fixed gantry predicting tomo IMRT	Model/DVH	44 Tomo/53 FG	Predicted vs clinical	Error bound	FG predict Tomo parotid mean dose: 92% cases within 10% error bound	
Wu et al.^47^ – IMRT predict VMAT	Case/DVH	12	Re‐planned VMAT vs clinical IMRT	Mean difference	Cord + 4 D.1 cc ‐3.7 Bstem D.1 cc ‐4.9 Larynx V50 ‐5.3 Brach plexus D.1 cc ‐1.6 Inner ear Dmean ‐4.4	
Tol et al.^12^	RapidPlan	15	Re‐planned vs clinical	Mean difference	Dmeans: Oral Cavity ‐2.7 Cparotid ‐1.2 Lower Larynx ‐5 Upper Larynx ‐5.7 Inferior PCM ‐5.8 Superior PCM –4.4 UES ‐3.5 Comp_swal ‐4.4	PTVb V95 0.5
Yuan et al.^13^	Model/DVH	20	Predicted vs clinical	Median difference of parotid D50 Sum of residuals of parotid DVH	Bilateral sparing cases: 0.34 Single‐side sparing cases: 2.2 Bilateral sparing cases: ‐0.002 Single‐side sparing cases: ‐0.08	
Schmidt et al.^61^	Case/voxel	10	Re‐planned vs clinical	Mean difference	Larynx Dmedian ‐3.6 Oral cavity Dmedian ‐5.5	Primary Dmax 1.3 Boost Dmax ‐1.3 HI ‐2.4 S_index ‐0.5
Tol et al.^18^	RapidPlan	20	Re‐planned vs clinical	Mean difference	Dmean:# Comp_sali ‐2.0 Oral cavity ‐3.6 Comp_swal ‐5.9	
Zhang et al.^42^	Model/DVH	148	Predicted vs clinical	Weighted root mean square error of DVH	~5.5%	

KBP, knowledge‐based planning; IMRT, intensity‐modulated radiation therapy; VMAT, volumetric arc therapy.

The difference direction is “KBP – Clinical”. Thus, negative values mean KBP value is smaller. Where differences are reported, only those that are statistically significant with a *P*‐value < 0.05 are listed in this table. The sign # indicates the significance is unclear and the sign ~ indicates the value is estimated from a graph.

**Table 7:** Performance of KBP on lung IMRT/VMAT (studies with 10 or more test cases)

Articles	Method type	Sample size	Validation target	Validation metrics	OARs	Target
Snyder et al.	RapidPlan	25	Re‐planned vs clinical	Mean difference	IMRT: Spinal Cord: D1.2 cc ‐0.5 D0.35 cc ‐0.8 D0.035 cc ‐1.0 VMAT: Esphagus: D0.035 1.1	IMRT: GI 0.66 VMAT: GI 0.25
McIntosh et al.	Case/voxel	17	Predicted vs clinical	Mean average difference over DVH of all ROIs	1.33^a^	
Faught et al.	RapidPlan	20 (functional‐guided plans)	Re‐planned vs clinical	Mean difference	Functional lung: V20 ‐1.8 Dmean ‐0.95 Lung‐GTV: V20 ‐1.6 Dmean ‐0.66 Esophagus: Dmean –2.6	

KBP, knowledge‐based planning; IMRT, intensity‐modulated radiation therapy; VMAT, volumetric arc therapy.

The difference direction is “KBP – Clinical”. Thus, negative values mean KBP value is smaller. Where differences are reported, only those that are statistically significant with a *P*‐value < 0.05 are listed in this table.

a Note that mean average difference (MAD) is not significant.

As these tables show, the validation sample size is relatively small with the prostate and H&N studies using 36 cases on average and lung studies using 21 cases on average. There are generally two types of validation studies, (a) comparing predicted dose metrics against those from the original clinical plans, and (b) comparing dose metrics of re‐planned cases, using the predicted dose parameters, against dose metrics of the original clinical plans. If the ultimate purpose of KBP is to produce treatment plans using the predicted dose parameters, the second type of comparison gives a more direct assessment of the KBP methods provided the implementation includes optimal use of the optimization engine.

The overall performance of KBP methods is difficult to evaluate because different studies use different metrics. For example, in prostate cancer KBP studies, we have seen various subsets of D90, D70, D50, D40, D35, D30, D25, D20, D17, D10, D1; D10 cc, Dmean; V100, V90, V75, V70, V65, V62, V56, V54.3, V50, V40, V39; gEUD, and NTCP to assess the dose distribution in bladder and rectum. While these endpoints are basically different ways to sample the DVH curve, most studies do not provide enough samples to allow reconstruction of the entire curve with reasonable accuracy. Furthermore, many studies do not report sufficient information. For example, some studies do not include prescription and planning constraints, while many studies report only the difference of dosimetric values. These factors make it difficult to carry out a meta‐analysis of the overall performance of KBP methods. This is especially true for H&N, lung, and other more complex cancer types.

For prostate cancer, we have found four KBP studies^27^, ^34^, ^35^, ^55^ that reported statistically significant reduction in mean dose to rectum and bladder after cases were re‐planned using the KBP methods. The pooled mean of reduction is 2.6 and 2.0 Gy for rectum and bladder, respectively. Incidentally, a more recent study of rectal cancer treatment published after the review articles were collected also resulted in an average reduction of 2.06 Gy in bladder mean dose.^79^

To gain further understanding of the overall performance of KBP in prostate cancer planning, we have developed a visualization scheme to provide a summary view of nine KBP prostate studies that compared re‐planned results with original clinically approved values.^20^, ^25^, ^27^, ^34^, ^35^, ^56^, ^57^, ^60^, ^63^ As mentioned previously, the challenge of summarizing results across all studies lie in two aspects: (a) the results are based on different sample points of the DVH curve and measure changes along different directions (e.g., one study may use D35 while another use V65); (b) some of the studies report only the differences in DVH point metrics (e.g., D35 is reduced by 1.5) without providing the original clinically approved values. The first issue makes it difficult to quantitatively compare results from different studies even though many DVH point metrics assess performance in similar areas of the DVH curve. While we cannot provide quantitative summaries, we can visualize the performance of different studies if we can define a base DVH curve, for example, by forming an average DVH curve of clinically approved plans. We can then identify the DVH points (e.g., D35 and V65) on the base DVH curve and display changes measured in KBP plans at these points to provide an overview of performance across all studies. We note that, since the study performance is based on DVH point metrics that are measured at multiple points along the DVH curve, it is important that we understand not only the extent of changes in the metrics, but also the spatial relationship of these metrics along the DVH curve. Unfortunately, the second issue makes it impossible to establish the base DVH curve and thus makes it difficult to visualize the spatial relationship of the various DVH point metrics, such as D35 and V65, that are reported in different studies.

In the proposed visualization scheme, we assume that the only common measure of performance reported across all studies is the difference between KBP re‐planned plans and clinical plans (i.e., KBP‐Clinical) for a set of DVH point metrics (e.g., D35 or V65). We overcome the second issue mentioned before by approximating the base DVH curve with a straight diagonal line connecting (0, 100) and (100, 0) in the dose‐volume space. We identify DVH point metrics, such as D35 or V65, along this approximate base DVH curve and display the differences (KBP‐Clinical) in these metrics as displacements along respective directions (e.g., horizontal displacements for D35 and vertical displacements for V65) to illustrate how well various KBP methods compare with the original clinical plans. Furthermore, we link the displaced points into approximate DVH‐change curves in the visualization scheme, so that the area below the diagonal line (i.e., the approximate base DVH curve) indicates the overall improvement over original clinical plans. In contrast, the curves that mostly lie above the diagonal line would indicate worse performance than clinical plans.

**Figure 3:**
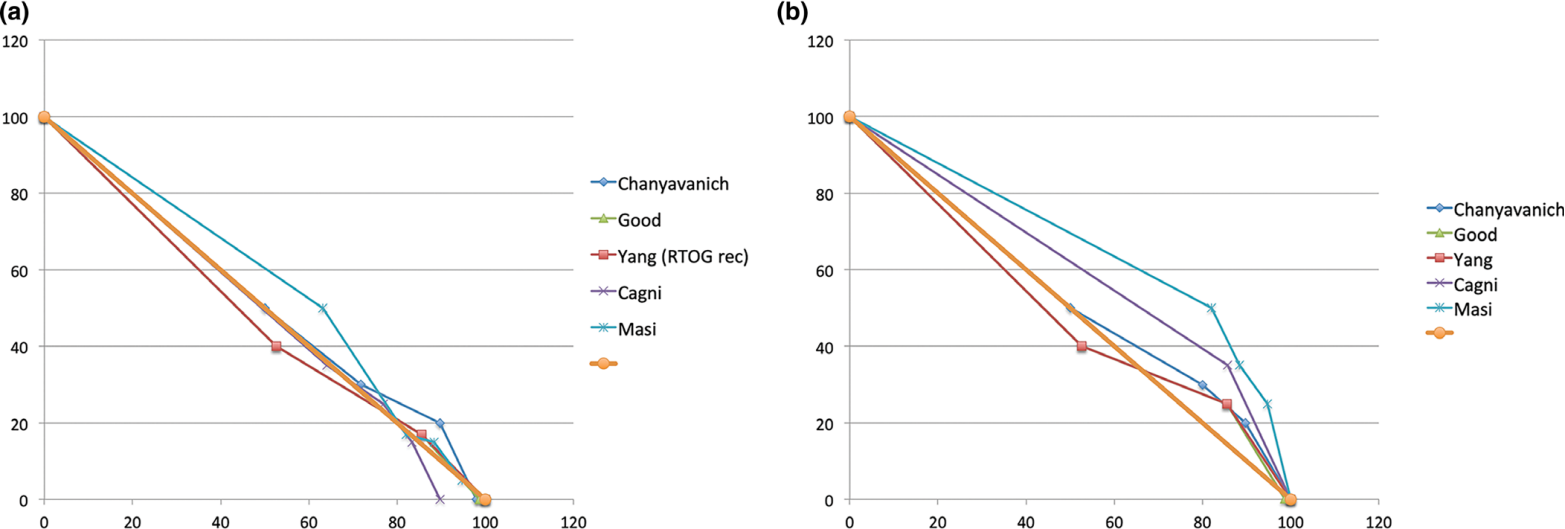
Prescribed dose‐volume constraints used for manual planning. (a) Rectum constraints; (b) Bladder constraints. Notice that in each case, the diagonal line (thick brown) is a reasonable first‐order approximation of the dose‐volume histogram curve. [Color figure can be viewed at wileyonlinelibrary.com]

We note that the choice of the straight diagonal line is somewhat arbitrary. The goal is to define a common framework to anchor the many different DVH point metrics from different studies and illustrate the relative region and extent of performance improvements. Incidentally, for rectum and bladder in prostate cases, we found that the diagonal line connecting (0, 100) and (100, 0) in the normalized dose‐volume space (percent volume and dose) is a good and simple proxy for the base DVH curves. As shown in Fig. 3, the prescribed dose‐volume constraints used for clinical planning in all nine published studies mostly lie just above the diagonal line. If we assume that most clinical plans achieve or slightly surpass the prescribed constraints, the base DVH curve of the clinical plans should be well approximated by the diagonal line to the first order.

**Figure 4:**
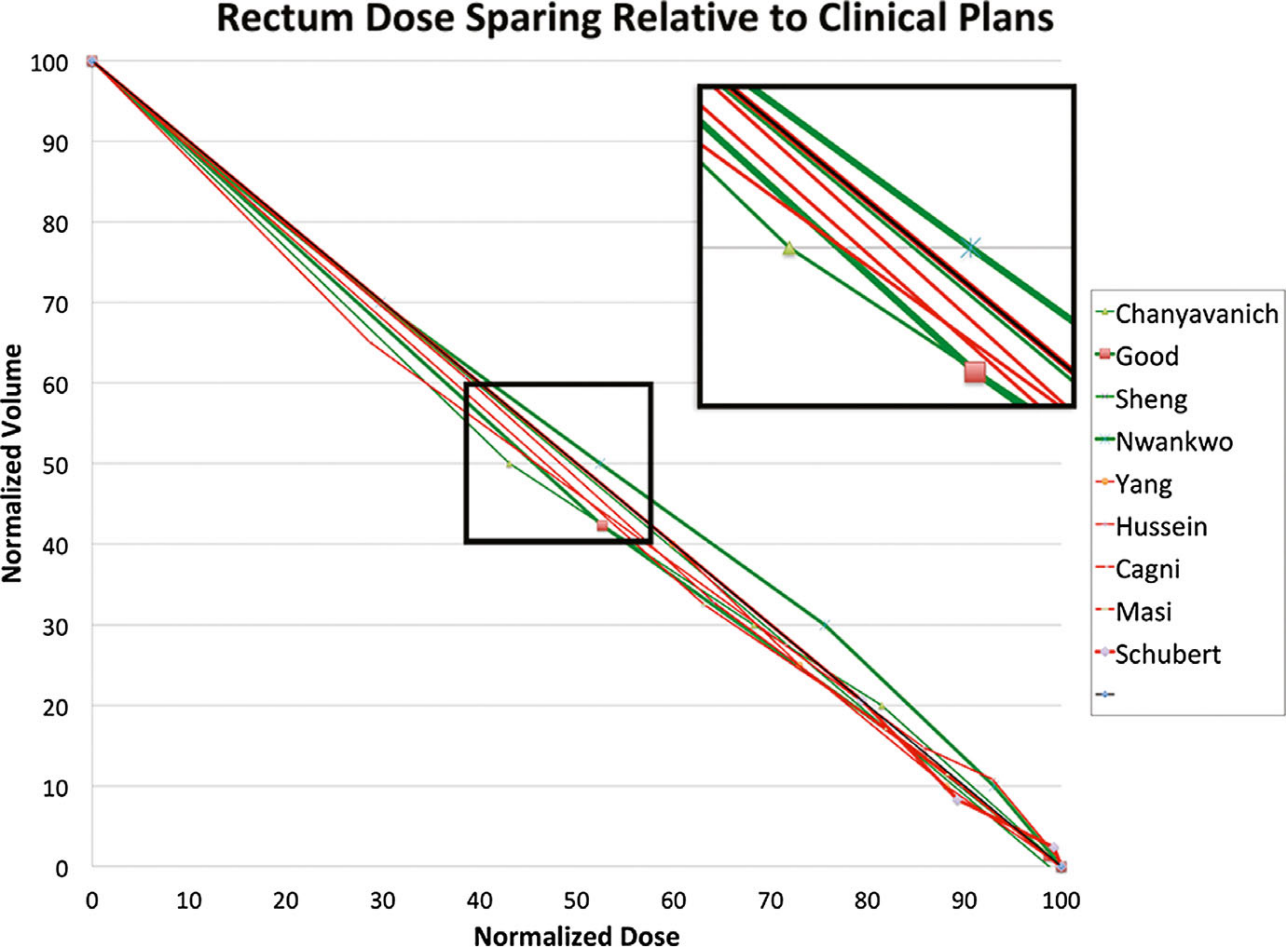
Visualization of knowledge‐based planning (KBP) method performance in rectum dose sparing. The thick diagonal line in black is the proxy dosevolume histogram (DVH) curve of clinical plans. The green and red DVH curves represent the approximated average performance of the re‐planned cases in nine KBP studies relative to the clinical plans. The green curves indicate case/atlas‐based methods while the red curves indicate model‐based methods. The thicker lines indicate studies with 30 or more sample cases. [Color figure can be viewed at wileyonlinelibrary.com]

**Figure 5:**
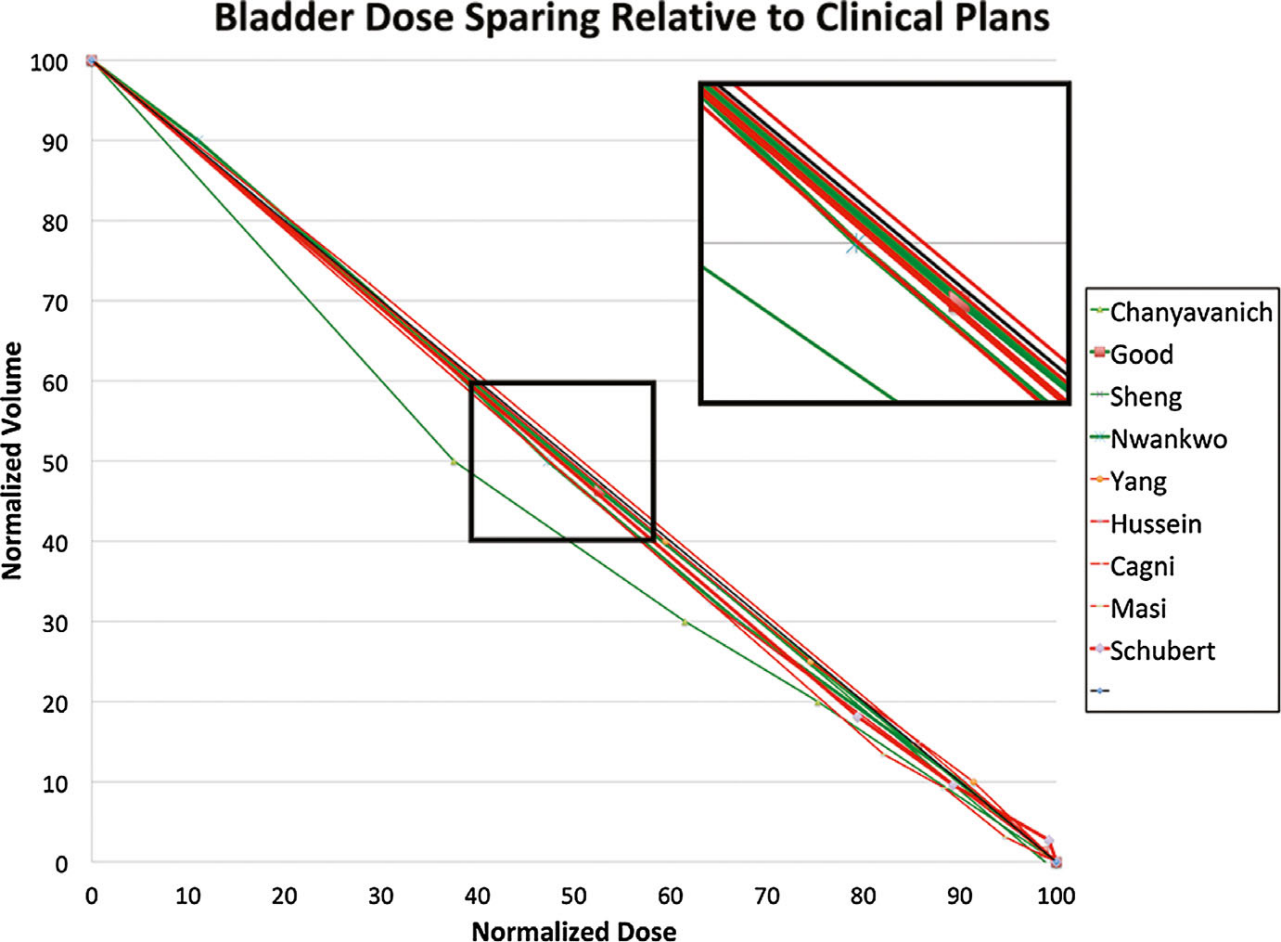
Visualization of knowledge‐based planning (KBP) method performance in bladder dose sparing. The thick diagonal line in black is the proxy dosevolume histogram (DVH) curve of clinical plans. The green and red DVH curves represent the approximated average performance of the re‐planned cases in nine KBP studies relative to the clinical plans. The green curves indicate case/atlas‐based methods while the red curves indicate model‐based methods. The thicker lines indicate studies with 30 or more sample cases. [Color figure can be viewed at wileyonlinelibrary.com]

Using the diagonal line as the approximate base DVH curve, we have plotted all the (KBP‐Clinical) differences of the DVH point metrics of the nine prostate studies in the same dose‐volume space after normalizing all values to the prescription dose. Figures 4 and 5 show the results for rectum and bladder, respectively. In these plots, we encoded the case/atlas‐based methods in green and the model‐based methods in red. Furthermore, we displayed the larger studies with 30 or more samples in thicker lines. As seen in these figures, most studies show an overall improvement in OAR sparing for both rectum and bladder although the improvement is mainly in the mid‐dose region. In the high‐dose region, the KBP methods perform about the same as the clinical plans. The mid‐dose region improvement is supported by significant mean dose reduction demonstrated in some studies. Moreover, the KBP approach and sample size do not appear to make a difference in performance although the case/atlas‐based methods (green curves) appear to have a larger variation.

The overall conclusion from all validation studies suggests that the KBP methods performed equally well on the target and mostly on par on the OARs with some improvements against the manual clinical methods. And some studies suggest that this is true especially for models learned from experienced planners’ datasets and applied to cases generated by either inexperienced planners or planners who are not experienced with a planning system.

Some studies^15^, ^20^, ^22^, ^28^, ^31^ have also compared the time and efficiency of KBP methods to the current manual planning process. In all cases, the KBP methods were faster and the improvement is more significant for more complex cases. Typically, timing comparison is between minutes of KBP methods and hours of manual planning process. However, these timing studies are preliminary because while the KBP methods can be precisely timed, the manual planning process is more difficult to measure objectively. Carefully designed prospective studies are needed to objectively assess the efficiency gains of KBP methods.

## 4 Discussion

The literature indicates that major growth in research efforts in the narrowly defined data‐driven KBP started in 2011 and has accelerated in the past a few years (See Fig. 2). We believe two factors contributed to this development. First, IMRT and related technologies that started in the turn of the century made the design of high‐quality treatment plans possible in this past decade. Second, the advent of IMRT over this period allowed large treatment centers to accumulate significant experience and a sizeable number of high‐quality plan data that enabled major progress in knowledge‐based research.

Most KBP studies have focused on prostate, head and neck, and lung cancers, although other types of cancer have received increasing attention in recent years. This trend will likely increase as more and more data and experiences are accumulated for the more complex or rare cancer types. Furthermore, although one case‐based decision support system has made use of clinical variables and a few others have incorporated trade‐off decisions into their models, most KBP methods are based on geometric and dosimetric parameters alone. It can be expected that more integral use of clinical, biological, and physics‐based parameters will further improve the performance of knowledge‐based approaches.

**Figure 6:**
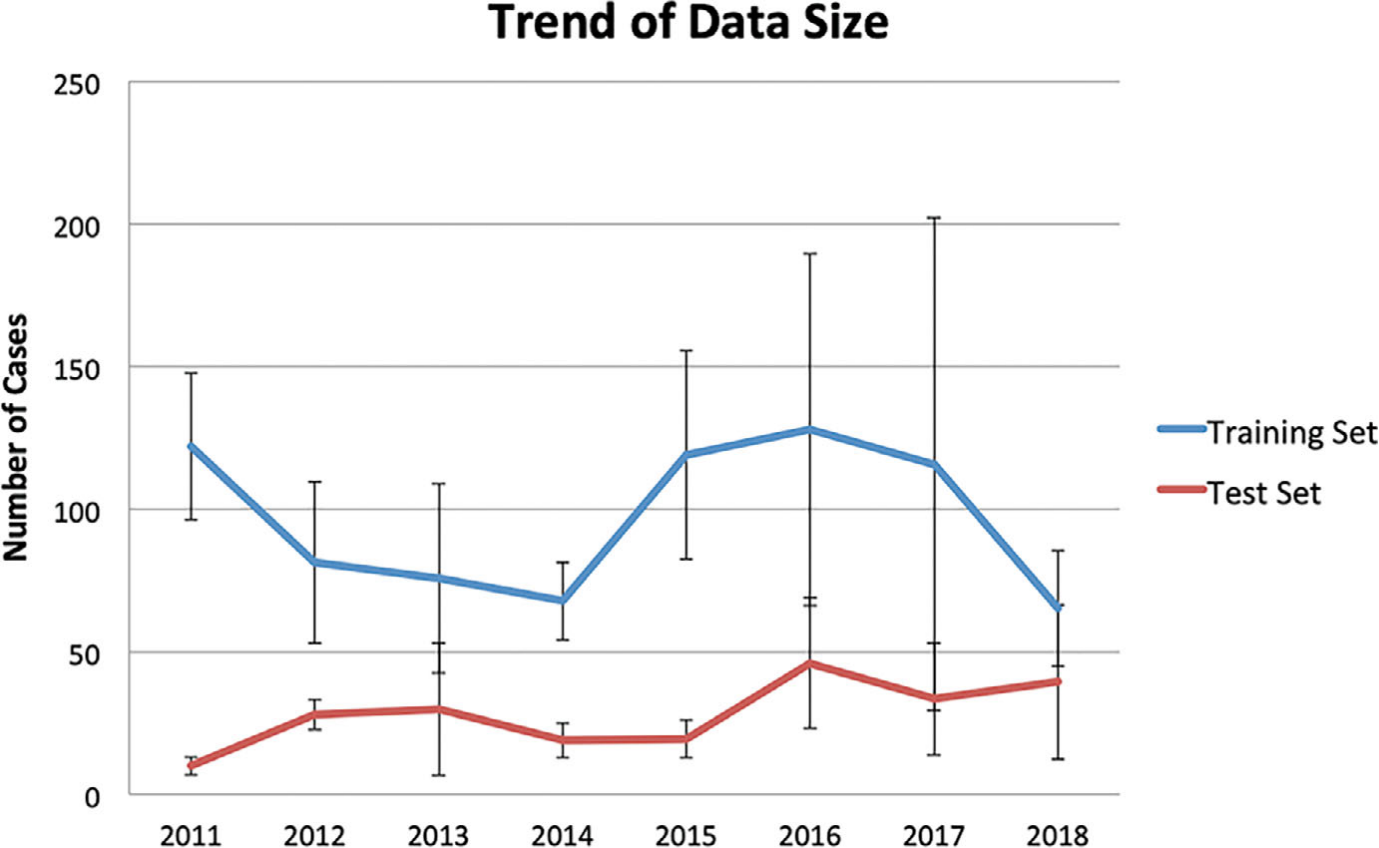
The size of datasets used for training and validating knowledge models. The error bars indicate standard deviation. Note that the large deviations in 2016 and 2017 are due to one significantly larger dataset. [Color figure can be viewed at wileyonlinelibrary.com]

Most studies are retrospective and use relatively small datasets. Figure 6 shows the average size of training and test datasets used in studies reported each year since 2011. We can see from this figure that the number of cases used for training and testing has not increased significantly in the existing studies. This is likely why multivariate linear regression has been quite successful in KBP modeling. More powerful machine learning models such as the artificial neural network will quickly overfit the small number of training samples and underperform the simpler regression methods. Even though the study by Boutilier^21^ suggests the number of cases required for training KBP models is relatively small, we believe these numbers are the result of simpler models. We should aim to develop larger training databases, so that we can use more sophisticated models to further improve the accuracy of KBP methods. It is probably unlikely that individual cancer centers will be able to boost sample size dramatically in short order. Thus, integration of cases from multiple centers or tapping into the national clinical trial datasets would help increase the sample size, although careful assessment of consistency across the cases is crucial. Furthermore, as the technology becomes mature enough, large‐scale prospective studies will be important to fully assess its performance in clinical applications.

Our reviews identify an important issue that the reported data and metrics used in validation studies are quite different and this is true especially for OAR sparing. These differences make it difficult, if not impossible, to pool accuracy results from multiple studies together in any statistically meaningful manner. The proposed visualization scheme allowed us to gain important insights into how well different types of KBP methods have performed in prostate cancer planning. However, this method is qualitative in nature. And its applicability to other types of cancer planning warrants further investigation. We believe it is critical to promote more standardized metrics and data reporting in future KBP studies, so that proper meta‐analysis can be applied to quantitatively estimate the performance of KBP methods. Without the strong evidences, clinical centers will not be able to objectively select and implement the most appropriate KBP methods.

In addition to larger scale and more standardized evaluation of data‐driven KBP methods, future research in this area will likely focus on more sophisticated modeling methods and more complex planning scenarios. Both directions will be enabled by the development of larger database of high‐quality clinical plans through integration efforts across consortium of institutions as well as accumulation of planning cases within individual institutions. Recent publications have shown promising results using complex nonlinear models such as convolutional neural networks to successfully predict voxel‐level dose in some cancer sites. Work has also begun to handle more complex cancer targets, more complex trade‐off decisions, as well as more complex treatment techniques. Beyond more complex and powerful models, the sophistication of modeling methods will also mean more advanced algorithms for learning, evolving, and integrating models. So far, data‐driven KBP has focused on building models in a batch mode, that is, learning from static datasets. As these models mature and are deployed in clinical use, another important research question will address how these models can be improved as new clinical cases are accumulated and new treatment techniques are developed.

As discussed in the Introduction, automatic planning methods represent another class of knowledge‐based methods for IMRT planning. These methods directly encode planning knowledge as rules and algorithms.^3^, ^4^, ^5^ A similarly large number of articles have been published in the past decade. A number of methods have also been implemented commercially. These methods were not included in this review because the central mechanisms are significantly different from the data‐driven KBP approaches. These methods deserve a separate review to properly understand the state‐of‐the‐art of its approaches and performance. Interestingly, a recent study by Wang et al.^80^ has applied a data‐driven KBP model to perform quality assurance of a commercially available automatic planning algorithm and demonstrated the potential of using KBP models to improve the performance of automatic planning algorithms. Another study by Babier et al.^81^ incorporated a KBP method into an automated planning method. We believe the combination of KBP models and automatic planning algorithms has a great potential to lead to further improvement of planning quality and efficiency in the future.

This review has examined KBP‐related papers since 2011. There are a few limitations. First, the review may have missed some papers due to use of a single Medline database and incomplete search strings. Second, the article selection criteria may have missed some relevant articles. For example, this review included only journal articles written in English language. Other publication venues and other languages may include valuable reports on KBP studies. Finally, as suggested in the publication trends numerous additional works have been published after the start of this project (e.g., Ref. 79, 81). These methods employ innovative strategies for using the KBP models to further improve plan quality and efficiency suggesting the need for a timely update of this review in the near future.

## 5 Conclusion

We have performed a systematic review of KBP methods and their validation results. A total of 73 articles are included in this review. These articles appeared in 16 journals and covered 21 cancer types and the number of publications has been increasing in the past years. We identified two major approaches to KBP, one based on cases and atlases, and the other based on statistical models and machine learning. In validation studies, both approaches have performed strongly. The KBP methods are generally equivalent to expert level planners in terms of plan quality but preliminary results indicate that they are significantly more efficient. These encouraging results suggest that clinical application of KBP to some cancer types such as prostate is achievable in the near future, ideally following additional validation studies using standardized metrics and prospective designs. Further development of KBP is warranted for more rare and more complex cancer sites. Larger datasets that are integrated across multiple institutions will be critical to achieve these more challenging goals.

## Conflicts of interest

The authors do not have relevant conflicts of interest to disclose.
